# Implementation and effectiveness of a multi-domain program for older adults at risk of cognitive impairment at neighborhood senior centres

**DOI:** 10.1038/s41598-021-83408-5

**Published:** 2021-02-15

**Authors:** Pei Ern Mary Ng, Sean Olivia Nicholas, Shiou Liang Wee, Teng Yan Yau, Alvin Chan, Isaiah Chng, Lin Kiat Philip Yap, Tze Pin Ng

**Affiliations:** 1 Geriatric Education and Research Institute (GERI), Singapore, Singapore; 2 Health and Social Sciences Cluster, Singapore Institute of Technology, Singapore, Singapore; 3 Duke-NUS Graduate Medical School, Program of Health Services and System Research, Singapore, Singapore; 4 KKT Technology Pte Ltd (Holmusk), Singapore, Singapore; 5 Neeuro Pte Ltd, Singapore, Singapore; 6 ProAge Pte Ltd, Singapore, Singapore; 7 Geriatric Medicine, Khoo Teck Puat Hospital, Singapore, Singapore; 8 Gerontology Research Program, Department of Psychological Medicine, National University of Singapore (NUS), Singapore, Singapore

**Keywords:** Neurological disorders, Disease prevention, Geriatrics

## Abstract

To address the paucity of research investigating the implementation of multi-domain dementia prevention interventions, we implemented and evaluated a 24-week, bi-weekly multi-domain program for older adults at risk of cognitive impairment at neighborhood senior centres (SCs). It comprised dual-task exercises, cognitive training, and mobile application-based nutritional guidance. An RCT design informed by the Reach, Effectiveness, Adoption, Implementation, Maintenance framework was adopted. Outcome measures include cognition, quality of life, blood parameters, and physical performance. Implementation was evaluated through questionnaires administered to participants, implementers, SC managers, attendance lists, and observations. The program reached almost 50% of eligible participants, had an attrition rate of 22%, and was adopted by 8.7% of the SCs approached. It was implemented as intended; only the nutritional component was re-designed due to participants’ unfamiliarity with the mobile application. While there were no between-group differences in cognition, quality of life, and blood parameters, quality of life reduced in the control group and physical function improved in the intervention group after 24 weeks. The program was well-received by participants and SCs. Our findings show that a multi-domain program for at-risk older adults has benefits and can be implemented through neighborhood SCs. Areas of improvement are discussed.

*Trial registration*: ClinicalTrials.gov NCT04440969 retrospectively registered on 22 June 2020.

## Introduction

The efficacy of large multi-domain interventions for dementia prevention, which include varying combinations of diet and/or medication, physical and cognitive exercises and other lifestyle modifications, have been widely discussed. Three large randomized controlled trials, (RCTs) namely the Finnish Geriatric Intervention Study to Prevent Cognitive Impairment and Disability (FINGER)^1^, the Multidomain Alzheimer Preventive Trial (MAPT)^2^ and the Dutch Prevention of Dementia by Intensive Vascular Care (preDIVA)^3^, which employed multi-domain interventions for dementia prevention have been conducted. Only the 2-year FINGER intervention showed improvement or maintained cognitive function in older adults at risk of dementia while secondary analysis of the MAPT study also showed some cognitive benefits to high-risk individuals. Additionally, a National Academies of Sciences, Engineering, and Medicine review reported support for cognitive training and increased physical activity in preventing dementia and cognitive decline^4^.

So far, no study has examined the factors that support the implementation of these multi-domain interventions. This lack of information on implementation methods impedes the research translation of effective interventions^5,6^. Evaluation studies that investigate the implementation are critical to consider acceptability, adoption, effectiveness, scalability and sustainability of the intervention in actual community settings^7^. Furthermore, comprehensive evaluation can help in determining the public health impact of interventions, aiding policy decision on resource allocation^5,6^.

With a rapidly ageing population^8^, dementia is an issue that Singapore directs national attention to. In 2016, the Health Ministry issued a National Innovation Challenge to fund pilot implementation of scalable preventive intervention programs that can be used safely to improve cognitive functioning in older adults^9^.

In response, this pilot project was conducted using a hybrid implementation-effectiveness design^10^ that concurrently (1) evaluated the implementation and (2) assessed the effectiveness of a multi-domain dementia-prevention intervention program among community-dwelling adults at risk for mild cognitive impairment and dementia in Singapore. The research team adapted the multi-domain intervention from the FINGER study and partnered business and community service providers to implement it at neighborhood senior centres (SCs). The RE-AIM framework (Reach, Effectiveness, Adoption, Implementation, and Maintenance) guided our evaluation^11^. This paper presents the findings of the study and discusses the program effectiveness and implementation.

## Methods

### Study design

The study consists of a 24-week program delivered to participants randomized into an intervention (IG) or wait-list control group (CG). Participants were randomized within each SC and remained compliant to their allocated groups. The IG participated in the program from 0 to 24 weeks while the CG continued with their usual routine. After the IG concluded their 24-week program and both groups completed post-program assessments, the CG also received the same program for ethical reasons. All interventions were instructor-led and access to the program was only granted while participants were active in the program.

Randomization was performed using computerized random numbers by a statistician who had no contact with participants. Participants and study assessors were blinded to their groupings.

The study obtained ethics approval from the National Healthcare Group Population Study Domain Specific Review Board (NHG DSRB Ref: 2017/00415). All research was performed in accordance with the relevant guidelines and regulations and in accordance with the declaration of Helsinki.

All participants gave informed consent before participating and no adverse event was reported.

### Participants

Sample size was calculated based on the recommendation by Billingham on the minimum number of participants required for a pilot trial^12^. Estimating a 50% attrition rate, a minimum of 100 participants per study arm was calculated as required for statistical significance between groups.

Participants were community-dwelling older adults aged 55 years and above at risk for cognitive impairment. Risk was determined using a risk scoring tool based on data from the Singapore Longitudinal Ageing Study (SLAS). Age, gender, education level, history of depression, satisfaction in life, hearing and presence of metabolic diseases such as diabetes, abdominal obesity, hypertension or abnormal blood lipid levels contribute to a maximum of 16 points (see Supplementary Table S1) (unpublished data). Older adults who scored 6 or more points were invited to participate in the study as that translated to a 10% risk of developing cognitive impairment in 5 years. Participants were excluded if they were those diagnosed with cognitive disorders or Parkinson’s Disease, were wheelchair bound, had total hearing or visual impairment, or had medical instructions prohibiting their participation in the program.

### Setting

Participants were screened and recruited from senior activity centres (SACs) and senior care centres (SCCs) by a study coordinator. SACs are located within residential estates and provide government subsidized space and activities for older adults to socialize with peers. SCCs are integrated care support centers that provide social daycare, dementia daycare, nursing and rehabilitation services to eligible older adults^13^. For conciseness, both SACs and SCCs will be described collectively as senior centers (SCs) henceforth. Potential participants were introduced to the program through an introductory talk conducted at various SCs. Interested parties were then screened for eligibility using the risk score.

### Intervention program

A bi-weekly program comprising cognitive training, physical-cognitive dual-task exercises and nutritional guidance was implemented by three local enterprises. The program comprised 48 sessions—31% physical-cognitive dual-task exercises and 69% cognitive sessions, of which 19% were based on small group activities and 50% were computerised cognitive training (CCT). Nutritional guidance was intended to be on-going via the application throughout the length of the intervention.

Cognitive training was delivered to participants in paper-based and computerized formats. Paper based games were designed and conducted by a psychologist to enhance memory, planning and executive functioning (ProAge, Singapore). Participants were placed in groups of 2–3 according to cognitive ability and had to complete gamified daily living tasks such as memorizing ingredients for specific recipes and route planning via public transport. The games had different levels of difficulties and progression to the next level was determined by the trainer. These games were conducted for 1.5 h once weekly for the first 12 weeks. In the computerized cognitive training (CCT), participants wore an electroencephalogram (EEG) headband^14^ that detects low frequency brain signals connected to a tablet while playing games that target attention, memory, decision making, spatial ability and cognitive flexibility via an application^15^ (Neeuro, Singapore). For example, in a game that trained attention, participants had to concentrate on guiding a helicopter to fly using EEG signals, without physically touching the tablet. When attention is lost or the mind is relaxed, the helicopter would land. These CCT games also had varying levels of difficulties and participants could decide if they wanted to progress. A trainer was present to facilitate each session. CCT sessions were 1 h once a week in the first 12 weeks, and 3 h a week (1 × 1 h and 1 × 2 h) in the next 12 weeks.

Physical training of moderate intensity was conducted in supervised groups of 6 to 10 participants (ProAge, Singapore). Moderate intensity was determined using the talk test^16^ where participants are able to talk, but not sing. Participants attended a 0.5 h and 1 h session on two different days of the week. The exercises were conducted by fitness instructors and incorporated physical-cognitive dual tasks, aerobic and resistance training (Table 1). Dual task activities included engaging participants in alternate dual language counting (i.e. English and Mandarin) during strength exercises (verbal fluency), and marching on the spot while moving upper body in the opposite direction of where the instructor points to (attention and motor coordination). Aerobic exercises involved coordination workouts with progressing difficulties as participants improved and low impact large muscle group workouts such as mimicking household chores (wiping windows, moping the floor) to mid-paced music. Resistance training was done using resistance bands and bodyweight exercises.

**Table 1:** Description of physical training protocol.

Phase	Physical training activity
Phase 1	1. Orientation and getting to know one another (social activities) 2. Dual tasking—Learning activities and moves 3. Aerobic—Building aerobic capacity (seated exercises for those who are weaker) 4. Strength—Seated body weight exercises to build foundation
Phase 2	1. Dual tasking—Progression of activities to increased difficulty 2. Aerobic—Progression to more standing related exercises 3. Strength—Standing body weight exercises with resistance bands
Phase 3	1. Dual tasking—Increased speed of activities (higher response time) 2. Aerobic—Aerobic exercise with functional movements 3. Strength—Functional movement with body weight and resistance bands

Progression of phase is dependent on participants in each class.

Nutritional guidance was delivered through a mobile application^17^ that enabled communications with a certified dietician online. Participants were taught to download and use the application on their smartphone before the start of the intervention and had free access to the application. Participants who had no smartphone were loaned one during program. They were asked to photograph and upload at least one main meal daily on the application as main meals usually consisted of more than one food item which would allow the dietician to give specific advice. The dietician would make relevant recommendations with the aim of encouraging healthier and affordable food choices, such as substituting white rice with brown rice. The application also contained access to online nutritional education modules, quizzes and games.

Once a month, the dietician gave a 1-h face-to-face nutritional talk that covered various health-related topics. These talks were also accessible to participants in the waitlist control group to maintain interest in the study.

### Outcome measures

Participants were assessed at baseline and 24 weeks (see Supplementary Table S2). The primary outcome was performance on the Repeatable Battery for the Assessment of Neuropsychological Status (RBANS)^18^. The battery consists of 12 subtests grouped into five domains—immediate and delayed memory, visuospatial/construction, language and attention. Total RBANS scores and domain scores were standardized into T-scores.

Secondary outcomes include the EuroQol EQ-5D-5L (EQ-5D, Rotterdam, The Netherlands), which were converted into index scores^19^ and reported with a self-reported health rating on the 0 to 100 Visual Analogue Scale (VAS), blood lipid panel (Cholestech LDX, Abbott, IL, U.S) and physical assessments. Physical assessments include the 2-min steps test, 30-s sit-to-stand test, and hand grip strength measured using a dynamometer. Physical assessments were conducted only for the IG during the first and last sessions of the program. Questionnaires with 5-point Likert scales and open-ended questions were also conducted. These questionnaires were administered verbally to participants, and online to implementers and centre managers. Open-ended questions such as “What kind of difficulties did you encounter when participating in the exercise sessions?” and “How do you think this program can be done differently?” allowed for the collection of free-text responses from all three groups.

### Statistical analysis

Baseline characteristics of participants in the intervention and control groups were analyzed using *t* tests or Wilcoxon rank sum tests for continuous variables, and χ^2^ tests for categorical variables.

Independent *t* tests and Wilcoxon rank sum tests were used to examine differences in cognitive, quality of life, and blood lipid panel outcomes between groups in complete case analysis. Within-group effects for cognitive, quality of life, and physical outcomes were analyzed using dependent *t* tests and Wilcoxon sign rank tests. Multiple comparisons were adjusted using Bonferroni correction.

### Qualitative analysis

Free-text responses were analyzed using inductive thematic analysis. Two researchers (PEMN, and SON) familiarized themselves with the text and made notes for potential codes. Potential codes were discussed until consensus on final codes. Subsequently, codes were consolidated into subthemes and then mapped into themes^20^.

### Evaluation

The RE-AIM framework measures used for evaluating the intervention and the data collection method employed is described in Table 2.

**Table 2:** Implementation evaluation measures, data sources and analysis method using RE-AIM framework.

Evaluation step	Description	Data source	Time point	Analysis method
Reach	The proportion of individuals willing to participate; and proportion who remained in the intervention	Recruitment records—signed ICF	Before the start of intervention	Number of successfully enrolled individuals/eligible individuals screened
		Attrition rate	Throughout the intervention	Number retained in intervention/number enrolled
Effectiveness	The short-term outcomes of the intervention; including subjective perceptions of participants	RBANS, EQ-5D, lipid panel data records	Before the start of the intervention and at 24 weeks after the intervention	The primary and secondary outcomes administered to all participants
		Physical fitness assessment records	At the start and last session of intervention	2-min step count, 30 s sit-to-stand, hand grip strength, ability to balance 10 s semi-tandem/tandem
		Participant questionnaires	After intervention at 24 weeks	Questionnaires with 5-point Likert-scale and free-text analysis
Adoption	Proportion of centres that agree to deliver the intervention	Enrolled centres record and site visit reports	Throughout the intervention	Number of successfully enrolled centres/total number of centres contacted
Implementation	Delivery of intervention components i.e. consistency and competency of community partners and adherence to protocol	Community partner questionnaires and attendance records	At the end of intervention and attendance record at the start of each session	Questionnaires with 5-point Likert-scale and free-text questions administered to participants and implementers
Maintenance	At the individual level: long-term effect of the intervention on participants At the organizational level: degree to which the intervention settings involved sustain over time	Participant questionnaires (self-report section on behavioural change)	At the end of intervention	Self-report section in the questionnaires administered to participants, centre managers and community partners
		Centre and community partner questionnaires (self-report section on willingness to continue with intervention)		

## Results

### Reach

Screening was performed by a study coordinator in 23 SCs, where 437 out of 672 older adults were eligible to participate. A total of 199 older adults (167 of them were females) were enrolled into the program study, with a mean age of 76.82 ± 8.97 years. Hence, the intervention reached 45.5% of the eligible population. Most of the participants (93.6%, n = 177) only had primary or no education, and 7.4% (n = 12) had at least secondary education. Mean cognitive impairment risk score of participants was 7.97 ± 1.13.

**Figure 1:**
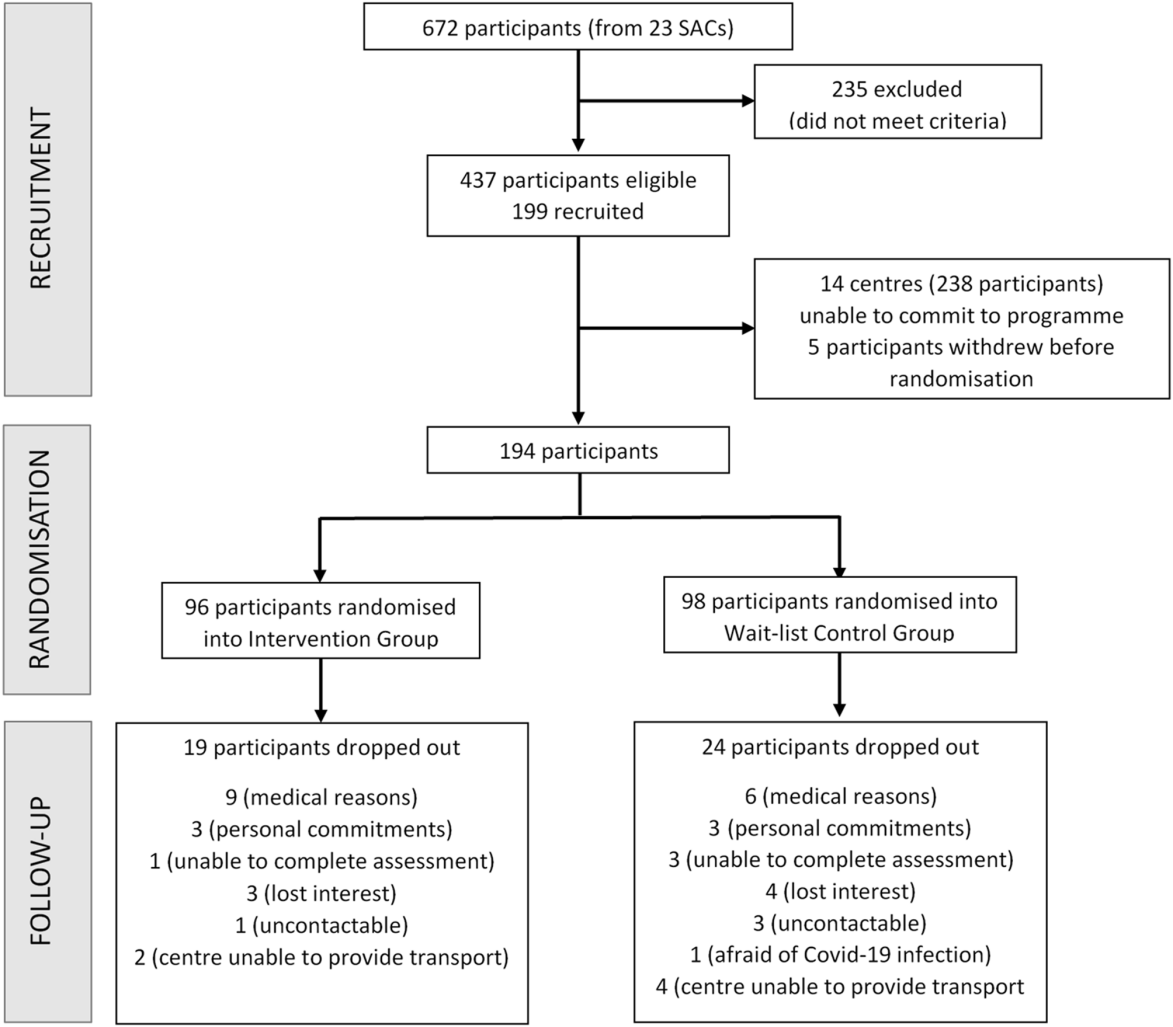
Participant flow diagram from initial contact to end of program.

Five participants withdrew from the study before baseline assessments (due to family objection, inability to commit to the schedule or language barriers). During the assessments, RBANS was conducted in either Mandarin or English. Due to their preference for dialects, a few individuals were unable to complete the RBANS and were considered as dropouts as the rest of the program required a basic understanding of English or Mandarin. The remaining 194 participants were randomized into the intervention (IG, N = 96) and control groups (CG, N = 98). During the study, a further 43 participants dropped out due to medical problems, loss of interest, personal commitments such as caring for grandchildren or death (unrelated to program participation) (Fig. 1).

### Effectiveness

There were no between-group differences in age, risk score, gender, education, total RBANS t-score, RBANS domains t-scores, EQ-5D index, EQ-5DVAS, and blood lipid panel measures at baseline (Table 3).

**Table 3:** Baseline characteristics of the participants.

Variable	IG		CG		p
	Mean ± SD	N	Mean ± SD	N	
**Characteristic**					
Age	75.61 ± 9.01	95	77.90 ± 8.84	96	.100
Gender		96		97	.705
Males	13.5%		15.5%		
Females	86.5		94.5%		
Education		97		92	.767
Below primary	92.8%		94.6%		
Above primary	7.2%		5.4%		
Risk score	7.92 ± 1.18	96	8.06 ± 1.06	97	.370
**Assessment**					
Total RBANS (T-scores)	51.38 ± 9.66	89	49.22 ± 10.12	94	.143
Immediate memory	51.43 ± 9.46	92	49.13 ± 9.94	96	.106
Visuospatial/constructional	50.84 ± 9.7	91	49.38 ± 10.33	95	.391
Language	51.34 ± 9.62	92	49.08 ± 10.04	96	.117
Attention	50.58 ± 10.08	92	49.62 ± 10.04	96	.498
**Delayed memory**					
Total cholesterol	174.7 ± 43.96	74	182.03 ± 39.42	80	.186
HDL	53.24 ± 14.18	74	56.70 ± 16.86	83	.114
LDL	99.14 ± 36.77	72	102.05 ± 35.01	79	.577
Triglycerides	118.36 ± 52.55	73	119.11 ± 57.33	81	.674
Glucose	110.15 ± 24.45	74	115.93 ± 47.34	82	.352
QoL VAS	79.39 ± 17.26	92	81.01 ± 17.47	97	.429
QoL Index	0.82 ± 0.21	93	0.83 ± 0.24	97	.319

*P* value indicates statistical difference between Intervention Group (IG) and Control Group (CG).

Blood test unit of measure are in mg/dL.

#### Between-group effects

There were no between-group differences in total RBANS score and domain scores after 6 months. There were also no between-group differences in quality of life measures and all blood parameters (Table 4).

**Table 4:** Cognitive, EQ-5D QoL and blood tests: effect of the multi-domain intervention programme within and between groups.

Variable	IG				CG				Group-time interaction
	N	Baseline	24 weeks	*P*	N	Baseline	24 weeks	*P*	*P*
Total RBANS	76	51.63 ± 9.71	51.07 ± 10.37	.219	72	50.04 ± 9.44	49.12 ± 9.63	.056	.238
Immediate memory	77	51.83 ± 9.23	50.22 ± 10.27	.055	71	50.46 ± 9.30	49.77 ± 9.84	.429	.785
Visuospatial/constructional	76	51.47 ± 10.03	50.7 ± 11.33	.454	71	50.18 ± 8.99	49.27 ± 8.45	.337	.391
Language	76	50.8 ± 9.44	51.08 ± 10.29	.759	72	49.87 ± 9.85	48.78 ± 9.73	.198	.164
Attention	77	51.06 ± 9.86	50.97 ± 10.04	.957	72	50.29 ± 9.65	48.98 ± 9.99	.008*	.206
Delayed memory	76	51.03 ± 10.16	50.38 ± 10.57	.365	72	50.31 ± 8.95	49.11 ± 10.27	.246	.462
Total cholesterol (mg/dL)	39	170.28 ± 47.41	176.72 ± 42.05	.107	42	177.1 ± 40.20	179.57 ± 39.06	.530	.765
HDL (mg/dL)	39	53.74 ± 16.03	51.95 ± 16.43	.839	43	55.58 ± 19.32	53.42 ± 13.89	.980	.801
LDL (mg/dL)	35	96.89 ± 40.87	107.31 ± 34.91	.013*	40	98.1 ± 35.18	100.4 ± 33.72	.562	.644
Triglycerides (mg/dL)	37	122.08 ± 56.54	114.41 ± 53.04	.369	42	130.17 ± 66.56	129.5 ± 92.72	.341	.358
Glucose (mg/dL)	38	108.95 ± 26.32	105.66 ± 19.18	.749	43	121.02 ± 58.90	121.63 ± 55.55	.776	.347
QoL VAS	78	79.67 ± 17.75	80.37 ± 18.22	.928	72	82.69 ± 16.00	74.43 ± 21.8	.000*	.071
QoL index	78	0.83 ± 0.21	0.84 ± 0.24	.075	72	0.85 ± 0.22	0.78 ± 0.29	.013*	.157

Data presented in mean ± SD.

*P < 0.05.

#### Within-group effects

There were no significant changes in total RBANS scores and immediate memory, visuospatial/constructional, language, and delayed memory scores in both the IG and CG from baseline to follow-up. The CG had significantly lower attention scores at follow-up (Median = 46.44) than at baseline (Median = 47.59), Z = 2.63, p = 0.0085. The CG also had significantly lower QoL VAS at follow-up (Median = 80) than at baseline (Median = 85), Z = 3.49, *p* = 0.0005, and significantly lower QoL index scores at follow-up (M = 0.78, SD = 0.29) than at baseline (M = 0.85, SD = 0.22), *t* = 2.54, *p* = 0.0133. LDL increased significantly in the intervention group following the intervention, Z =  − 2 .97, *p* = 0.003, with baseline median LDL at 93 mg/dL and follow-up median LDL at 100.5 mg/dL (Table 4).

The IG improved significantly in the 2-min steps test and both left and right handgrip strength following the intervention (Table 5).

**Table 5:** Physical performance test: effect of the multi-domain intervention programme within the intervention group.

Variable	N	Baseline	24 weeks	*P*
Steps test	57	54.47 ± 23.65	62.77 ± 27.07	.0018*
Chair stand	69	14.09 ± 5.68	16.75 ± 6.54	< .0001*
Handgrip strength (R)	74	16.36 ± 5.61	18.15 ± 5.53	< .0001*
Handgrip strength (L)	74	15.63 ± 5.31	17.02 ± 5.05	.0002*

Data presented in mean ± SD. *P* value indicates statistical difference within Intervention Group (IG).

Subthemes that emerged from participants’ free-text responses included physical, cognitive, and psychosocial benefits through participation in the program. Participants felt “more toned”, had “more strength”, and “walked faster,” and did not need to take certain chronic illness medications anymore. Effectiveness of the exercises in improving physical fitness achieved a mean score of 4.21 ± 0.63. Participants felt that the cognitive games improved their alertness, allowed them to make new friends, and boosted their self-confidence. Small group activities and CCT games achieved a mean effectiveness score of 3.93 ± 0.46 and 3.97 ± 0.45, respectively (Table 6).

**Table 6:** Responses from IG participant questionnaire (n = 70) [maximum score of 5].

Questionnaire items	Score
**Dual-task exercise**	
How often did you attend the group exercises in a month?	4.56 ± 0.82
Do you find the exercises easy to follow?	3.96 ± 0.71
Do you feel that the exercise sessions were useful in improving your physical fitness?	4.21 ± 0.63
**Small group activities**	
How often did you attend the games/activity session in a month?	4.60 ± 0.87
Do you find the games/activities easy to follow?	3.53 ± 0.89
Do you feel that the games/activities were useful in improving your cognition?	3.94 ± 0.46
**Computerised cognitive training**	
How often did you attend the cognitive classes in a month?	4.44 ± 1.00
Do you think the cognitive classes were easy to follow?	2.79 ± 0.93
Do you think the cognitive classes were useful in improving your cognition?	3.97 ± 0.46
**Nutritional guidance**	
How often did you use the Glycoleap handphone application in a month?	1.28 ± 0.76
Do you think the Glycoleap handphone application was user-friendly?	2.32 ± 1.28
Do you think that having a dietician was helpful in making healthier food choices?	3.27 ± 1.23
Have your eating habits changed after the programme?	3.58 ± 0.62

Data presented in mean ± SD. Questions were based on 5-point Likert scale where lower scores indicate negative responses and higher scores indicate positive responses.

### Adoption

During the recruitment phase which spanned over a year, recruiters contacted 103 SCs via email, of which 39 centres (38%) responded and 23 SCs were screened. The program was eventually adopted by 9 centres (8.7%), two of which were formed through combining themselves with smaller affiliated centres in the vicinity. The other 14 SCs could not take up the program despite interest as they were unable to accommodate the study within their schedule or lacked eligible participants to form a sizeable group—an estimate of 30 participants per centre was deemed as most practical for training and doing group activities.

77 participants from IG completed the intervention and both assessments and 74 from CG completed both assessments. On average, IG attendance for the dual-task physical exercises, small group activities and CCT sessions was 75.78 ± 19.13%, 78.90 ± 20.25% and 70.99 ± 23.77% respectively. Only 15% of the IG used the nutritional application consistently although participants were taught usage one-on-one. Smart phones were loaned to four participants, but were returned unused. Others who did not own phones declined to take up the loan.

SCs reported space constraint and difficulties in encouraging participation. Space to accommodate an average of 10 participants was required to conduct group physical exercises. However, SCs are located in the void decks of public housing flats and some have limited indoor floor area for group physical exercises. The group exercises for larger groups are conducted outside the SAC premises. The lack of space is compounded by multiple simultaneous activities at the centres, such as board games and handicraft sessions. Most centers made phone calls to remind participants to attend classes.

### Implementation

When approval and support from management of each centre was obtained, the program was implemented within 2–3 months. The first session started within 2 weeks after baseline assessment. Researchers made random visits to observe the sessions and ensure that the program was conducted according to schedule and protocol.

There were no major deviations from the protocol other than the nutritional component. Due to participants’ unfamiliarity with the nutritional application and IT in general, and the lack of WiFi connection at home, participation rate was very low—86% reported that they did not use the application. Participants reported difficulties in all stages of using the application—logging in, staying logged in, taking and sending photographs and conversing with the dietician via the application, or remembering to take photographs.

To mitigate the low usage rate, dieticians made phone monthly calls (15–20 min) to participants. Despite this, some participants remained uncontactable. Only 16 (20%) participants responded to all six consultation phone calls, 42 (52%) responded to at least three calls and 61 (76%) responded to at least one call. Participants reported difficulties with phone calls due to hearing problems, phones switched off or fear of calls from strangers. The dietician also suggested that on some occasions, there was no one at home.

Other minor deviations were reported by exercise trainers who had to conduct physical assessments at the program’s first session initially, resulting in the reduction of exercise time from 1 h to less than 15 min. However, this was not deemed to have any impact on the outcome of the data. To mitigate this, subsequent physical assessments were performed by researchers.

Implementers suggested that the lack of SCs and their own manpower introduced additional challenges as many participants had mobility limitations (e.g. slow gait or could not stand for long). Implementers found that if they had to assist these participants to use the bathroom or retrieve items, other participants would be sometimes neglected. As some of the physical exercises were considered strenuous to participants with health conditions, implementers felt that the medical conditions of participants may not have been adequately screened by the SCs using PAR-Q assessment and had to make additional assessments to identify participants who required additional attention during training.

The CCT implementer reported that tiredness and restlessness from exercise sessions prior to the CCT sessions and insufficient training time were reasons for less than optimal engagement. The lack of space in some SCs had also been cited as a constraint.

Overall, both participants and centre managers had positive feedback for trainers and for the program, suggesting that the program was well delivered. Participants’ overall satisfaction with the program was 3.90 ± 0.80 (out of 5) and 94.2% of participants would recommend the program to their relatives and friends. Participants had positive comments for the program and trainers for all components of the program. The program was “fun” and “novel’, and trainers were described as “helpful”, “accommodating”, “lively”, “funny”, and “patient”. Similarly, centre managers’ overall satisfaction with the program was 4.67 ± 0.47 (out of 5), and 100% of them would recommend the program to other centre managers.

The cost of the program was estimated by the implementers to be SG$620 per participant, not including the cost of manpower and a tablet needed for cognitive training. When participants were asked how much they were willing to pay for the whole program, out of 70 who answered, 17.4% were willing to pay less than $10, 21.7% less than $50 and 7.2% less than $100. More than 50% were not willing to pay or were unsure. Reasons included being on financial support or not being able to valuate the program. As the program was conducted in SCs, there was no cost for renting of spaces and most participants were not required to travel, other than to the centres they were already visiting almost daily.

### Maintenance

All centres who completed the questionnaire expressed interest to continue with the program. To date, two centres have approached one of the implementation partners to discuss the possibility of holding health talks for their members. There were no other enquiries or enrolment for the other components. This could be due to cost—most SCs rely on government support and private donations and will have limited funds for additional activities. Five centres have yet to complete the full program due to COVID-19 related mandatory suspension of centre activities.

48 out of 70 IG participants (68.5%) reported that they intended to continue doing the exercises they had learnt. Nine participants did not want to continue the program beyond the study, five only wanted to continue the exercise component of the program, and 54 wanted to continue the entire program beyond the study.

## Discussion

Overall, the program was sufficient to elicit improvement in physical but not cognitive performance, and was well received by participants, implementers, and community partners. Many of the IG participants, 65% of whom were above 75 years, tolerated the dual-task physical exercises well. This could be because training was conducted in smaller groups and personalized. The social aspect associated with engaging in small group activities could have facilitated positive outcomes of the intervention, as a few participants reported new friendships as a result of the program. Other studies have reported that group settings and mutual support may elicit improved physical fitness^21^, more sustained program participation^22^ and better quality of life^23^. While it was beyond the scope of the study to ascertain the mechanism through which a multi-domain intervention results in positive effects for its participants, there is sufficient evidence that physical exercise alone can maintain cognitive function^24,25^ and quality of life^26,27^ in older adults and should continue to be encouraged.

Several studies have highlighted the beneficial effects of CCT in older adults^28–31^. With application of technology and suitable community-based programs, new cognitive training platforms can easily be disseminated to the older population. Participants need not have IT skills to benefit from CCT^32^. However, the delivery and approach of CCT applied in this study might not have been suitable for this group of participants, who, given their age and lower education levels, might have not been previously exposed to tablet computers. The sudden and long exposure could have compromised on engagement despite small class sizes. IG participants’ perceived report on whether CCT sessions were easy to follow was rated an average of 2.79/5, compared to the paper-based cognitive classes which achieved a mean rating of 3.53/5. Mean attendance was also lower for CCT sessions (67.6%) compared to the paper-based cognitive sessions (77.7%). The Singapore government recognises the potential of telehealth to deliver support and have increasingly enabled and encouraged older adults to use them^33,34^. However, as acceptance is a prerequisite to adoption^35^, the type of technology applied should not only be tailored to match their needs^36^ but should also be perceived to be useful and easy to use.

The challenges with technological devices also compromised the delivery of the nutritional component of the study. While pen-and-paper meal diaries might have led to more consistent dietary data, a mobile application was used for its ability to facilitate real-time and consistent interaction with dieticians. However, participants gave low scores for the frequency and usefulness of the nutritional application (Table 6) and struggled to use the application. Participants who did not own smart phones refused to be loaned one for fear of losing or using it or simply did not use the application. Reasons for this reluctance might include personal anxiety, limited self-confidence^37^ and fear of misuse of technology—that the device/application might not perform as desired or might compromise their privacy. Inaccessibility issues could also be due to financial or physical inaccessibility—both of which could be related to lack of support and assistance^38^. Indeed, 30% of the participants in this study lived alone or with another elderly spouse and lacked social support.

Additionally, some participants found it hard to switch to healthier dietary options as they dined with family who resisted change. Others relied on food donations or could not afford healthier options. About 60.4% reported no change to their diet for the duration of the intervention. However, participants perceived the advice from the dietician as helpful, rating its usefulness an average of 3.3/5. Regular access to nutrition services will empower older adults in making healthier choices and they will benefit more from sessions that involve active participation—such as visiting supermarkets (either actual or virtual guided tours) or hands-on meal preparation cum eat together sessions.

Temporal factors should also be considered during implementation. The larger multi-domain studies had interventions that were minimally 1 year^1–3^ whereas this intervention duration of 6 months could be barely sufficient to elicit significant cognitive effects. Additionally, the intensity of CCT sessions in this intervention may be of moderate intensity but appeared too cognitively demanding for participants. Coupled with distractions and tiredness after the physical training, most of them found some sessions too challenging for them to try to improve on their game scores and levels. Some might also have decreased motor and visual abilities^39,40^, sensitivity to glare^41^ while interacting with screens and devices or cognitive changes which are reflected in decreased perception, memory, processing speed, attention or inability to ignore irrelevant stimuli and thoughts^42,43^. As such it is possible that insufficient time was allocated to the CCT sessions. Extra time for settling in, familiarization and orientation of tablet functions and games and frequent short breaks may be needed for these participants.

While the intervention may have reached the intended group of community dwelling older adults who are at increased risk of cognitive impairment, less than half of eligible participants agreed to participate in the study. Reasons included (1) denial of higher-risk status or the stigma of being associated with dementia, (2) reluctance to commitment to a training cum research program with additional administrative requirement (forms to read, consents to sign and procedures to follow i.e. being randomized). Furthermore, as recruitment was dependent on the active participation of older adults at their respective SCs, the program was not able to reach socially isolated adults who are at higher risk for dementia but do not participate actively at the centres. This group might benefit more from the program than older adults who are already physically, mentally and/or socially active. While there are local initiatives to reach this segment of older adults, it is also crucial to raise public awareness of dementia and availability of stimulating programs.

Overall, the program was well received by both participants and their centres. SCs in Singapore likely need additional funding support to contact other providers to deliver targeted and effective preventive services. In our study, the centre managers facilitated the recruitment of participants and reminded them to turn up for the program and assessments. The engagement and involvement of community partners at all (the planning, pre- and post-implementation) stages are crucial towards improving effectiveness and implementation outcomes. This also facilitates accountability, ownership and may increase likelihood of continued adoption and maintenance of a program.

While our study used a variety of data sources to evaluate both the effectiveness and implementation of a community-based program and provided valuable local insights that could help bridge the research-practice gap in dementia prevention, the generalizability of results is impacted by the small sample size and lack of follow-up (partly due to COVID-19 related suspension). Another limitation is that the physical assessment was not conducted on the control group which prevented between-group comparison from being made. Table 7 summarizes the key findings of the evaluation and areas of improvements for the program.

**Table 7:** Key findings of the multi-domain intervention evaluation.

Key success factors	Improvement factors
Increased out-reach to older adults through Senior Centres Support from centre managers enabled smooth implementation of programme Availability and willingness of centres to incorporate intervention into their programme schedule Willingness of participants to be randomised Programme fully implemented by three partner enterprises in the community Physical-cognitive dual-task exercises of moderate intensity were tolerated and enjoyed by participants with benefits to physical performance Small group mental activities which simulate daily living activities enjoyed by participants	Reaching out to target older adults (socially isolated, lesser engaged) Temporal considerations Adequate time for CCT factoring in frequent breaks and learning curves Increasing sessions on small group activities (non-technology related) Longer intervention period i.e. > 24 weeks Addressing technology confidence and knowledge Involvement of community partners in earlier stages of planning

## Conclusions

Our study employed an effectiveness-implementation hybrid design to evaluate the effectiveness and implementation of a multi-domain program in the community. The overall positive feedback from participants, SCs, and implementers suggests that such a program can be feasibly implemented in the community. Longer term implementation research is needed to understand how to achieve more effective and sustained benefit for such a program to improve or maintain cognitive health in vulnerable older adults.

## Supplementary Information

Supplementary Tables.

## Data Availability

The study datasets used for analyses are available from the corresponding author upon reasonable request.
