# Supplementary material for: Implementation and effectiveness of a multi-domain program for older adults at risk of cognitive impairment at neighborhood senior centres
Source: Sci Rep. 2021 Feb 15;11:3787. doi: 10.1038/s41598-021-83408-5 (PMC7884402; doi:10.1038/s41598-021-83408-5)
Supplement: Supplementary file 1 — Supplementary Tables. [file 41598_2021_83408_MOESM1_ESM.pdf]

Title: Implementation and effectiveness of a multi-domain program for older adults at risk of cognitive impairment at neighborhood senior centres

Authors: Pei Ern Mary Ng[1] , Sean Olivia Nicholas[1] *(co-first authors)*, *Shiou Liang Wee[1,2,3], Teng Yan Yau[4], Alvin Chan[5], Isaiah Chng[6], Lin Kiat Philip Yap[1,7], Tze Pin Ng[1,8]

Affiliations:

[1] Geriatric Education and Research Institute (GERI), Singapore

[2] Health and Social Sciences Cluster, Singapore Institute of Technology, Singapore

[3] Program of Health Services & System Research, Duke-NUS Graduate Medical School, Singapore

[4] KKT Technology Pte Ltd (Holmusk), Singapore

[5] Neeuro Pte Ltd, Singapore

[6] ProAge Pte Ltd, Singapore

[7] Geriatric Medicine, Khoo Teck Puat Hospital, Singapore

[8] Gerontology Research Program, Department of Psychological Medicine, National University of Singapore (NUS), Singapore

* Corresponding Author: Wee Shiou Liang, weeshiouliang@gmail.com, +6565923261

Supplementary Table S1: Risk score for cognitive impairment

Variables	Score
Age 65 – 74	1
Age ≥ 75	2
Female	1
Primary or no education	3
History of depression or ≥ 5 depressive symptoms	1
Not very satisfied with life	1
Hearing impairment	2
1 – 2 metabolic diseases*	2
3 – 4 metabolic diseases*	3

*Hypertension, abdominal obesity, abnormal blood lipid levels, diabetes or pre-diabetes

Supplementary Table S2: Assessments at baseline and 24 weeks

Assessment	Baseline	24 weeks
**Cognitive**		
RBANS	x	x
**Quality of life**		
EQ-5D	x	x
**Physical***		
Hand grip strength (kg)	x	x
Balance (10s)	x	x
Chair stand (30s)	x	x
Steps test (2m)	x	x
**Blood test (mg/dL)**		
Total cholesterol	x	x
HDL and LDL Cholesterol	x	x
Triglycerides	x	x
Glucose	x	x
**Questionnaires**		
Participants		x
Centre managers		x
Implementers		x

*Physical assessment was only conducted on the first and last session of the programme for each group.
